# Supplementary material for: Rapid spread and population genetics of Aedes japonicus japonicus (Diptera: Culicidae) in southeastern Europe (Croatia, Bosnia and Herzegovina, Serbia)
Source: PLoS One. 2020 Oct 29;15(10):e0241235. doi: 10.1371/journal.pone.0241235 (PMC7595422; doi:10.1371/journal.pone.0241235)
Supplement: S1 Table — (DOCX) [file pone.0241235.s001.docx]

**S1 Table.** Study locations, collection approaches and outcome.

| Number in Fig. 1 | Location  [place/country] | GPS coordinates | Altitude [m a.s.l.] | Collection date(s) | Collection method | Type of collection site/area | Number of breeding sites/traps sampled (no. positive) | No. of *Ae. japonicus* collected ^#^ |
| --- | --- | --- | --- | --- | --- | --- | --- | --- |
| 1 | Klikun, CRO | 45.281667, 17.716667 | 325 | 5 Jul 2017 | Dipper | Rock pool | 1 (1) | 1 |
| 2 | Laze Prnjavor, CRO | 45.285556, 17.702222 | 304 | 5 Jul 2017 | Dipper | Bathtub | 1 (1) | 33 |
| 3 | Laze Vasine, CRO | 45.306667, 17.720833 | 347 | 5 Jul 2017 | Dipper | Pond | 2 (1) | 1 |
| 4 | Jankovac, CRO | 45.521389, 17.685000 | 493 | 6 Jul 2017 | Dipper | Pond | 1 (1) | 2 |
| 5 | Slatinski Drenovac, CRO | 45.551944, 17.709167 | 184 | 6 Jul 2017 | Dipper | Barrel | 3 (1) | 4 |
| 6 | Feričanci, CRO | 45.517778, 17.975833 | 158 | 18 Aug 2018 | Dipper | Flower vase | 9 (1) | 6 |
| 7 | Slatina, CRO | 45.420814, 17.420436 | 103 | 24 Aug 2017 | Ovitrap | Tyre service | 3 (1) | 4 |
| 8 | Orahovica, CRO | 45.532853, 17.879537 | 183 | 17 Aug 2017 | Ovitrap | Tyre service | 3 (2) | 20 |
| 9 | Belo Selo, CRO | 45.194512 14.442279 | 797 | 11 Jul 2017 | Dipper | Tyre | 1 (1) | 4 |
| 10 | Delnice 1, CRO | 45.405556, 14.798611 | 712 | 11 Jul 2017 | Dipper | Bathtub | 1 (1) | 1 |
| 11 | Zamost, CRO | 45.530278, 14.686667 | 234 | 11 Jul 2017 | Dipper | Barrel | 1 (1) | 1 |
| 12 | Sungerski Bukovac, CRO | 45.345000, 14.779722 | 776 | 11 Jul 2017 | Dipper | Barrel | 10 (1) | 1 |
| 13 | Delnice 2, CRO | 45.392500, 14.796111 | 698 | 12 Jul 2017 | Dipper | Tyre | 1 (1) | 3 |
| 14 | Fužine, CRO | 45.307778, 14.715833 | 739 | 12 Jul 2017 | Dipper | Tyre | 1 (1) | 2 |
| 15 | Lič, CRO | 45.273889, 14.718333 | 706 | 12 Jul 2017 | Dipper | Barrel | 8 (1) | 11 |
| 16 | Lič-Bribir, CRO | 45.220278, 14.806389 | 896 | 12 Jul 2017 | Dipper | Barrel | 2 (1) | 4 |
| 17 | Kupjak 1, CRO | 45.390000, 14.884722 | 778 | 12 Jul 2017 | Dipper | Tyre | 4 (4) | 12 |
| 18 | Kupjak 2, CRO | 45.388611, 14.885278 | 776 | 12 Jul 2017 | Dipper | Tyre | 1 (1) | 1 |
| 19 | Skrad, CRO | 45.427778, 14.903611 | 702 | 12 Jul 2017 | Dipper | Bathtub | 1 (1) | 13 |
| 20 | Baške Oštarije, CRO | 45.537500, 15.147778 | 921 | 1 Aug 2017 | Dipper | Rock pool | 1 (1) | 1 |
| 21 | Kuterevo, CRO | 44.823333, 15.141944 | 529 | 2 Aug 2017 | Dipper | Barrel | 1 (1) | 5 |
| 22 | Macelj, CRO | 46.443611, 16.441389 | 392 | 13 Jun 2017 | Ovitrap | Border crossing | 6 (2) | 19 |
| 23 | Konjščina, CRO | 46.100000, 16.282778 | 175 | 12 Jun 2017 | Ovitrap | Cemetery | 3 (1) | 14 |
| 24 | Ljuba, SRB | 45.174166, 19.364722 | 245 | 21 Aug 2018  15 Sep 2018 * | Ovitrap | Border crossing | 5 (1)  5 (1) | 7  1 |
| 25 | Odžak, BiH | 45.013173, 18.326002 | 99 | 7 Jul 2017 ** | Ovitrap | Courtyard | 5 (1) | 21 |
| 26 | Brčko, BiH | 44.806744, 18.806744 | 80 | 9 Aug 2017 ** | Ovitrap | Courtyard  Border crossing | 4 (1)  1 | 1 |
| 27 | Bučje, CRO | 45.468529, 17.354803 | 267 | 4 Jul 2017 | CDC trap | Forest | 1 | 0 |
| 28 | Glavica, CRO | 45.454351, 17.430256 | 298 | 4 Jul 2017 | CDC trap | Forest | 1 | 0 |
| 29 | Kamenska, CRO | 45.446842, 17.473517 | 270 | 4 Jul 2017 | CDC trap | Forest | 1 | 0 |
| 30 | Novo Zvečevo, CRO | 45.548833, 17.515266 | 483 | 4 Jul 2017 | CDC trap | Fishpond | 1 | 0 |
| 31 | Prijevoj, CRO | 45.559125, 17.503227 | 494 | 4 Jul 2017 | CDC trap | Forest | 1 | 0 |
| 32 | Striježevica, CRO | 45.468768, 17.506175 | 442 | 4 Jul 2017 | CDC trap | Forest | 1 | 0 |
| 33 | Crkveni Vrhovci, CRO | 45.288325,17.651208 | 353 | 4 Jul 2017 | CDC trap | Courtyard | 1 | 0 |
| 34 | Gradski Vrhovci, CRO | 45.300496, 17.638191 | 286 | 4 Jul 2017 | CDC trap | Courtyard | 1 | 0 |
| 35 | Jagodnjak, CRO | 45.311267, 17.669017 | 263 | 4 Jul 2017 | CDC trap | Forest | 1 | 0 |
| 36 | Požeška Koprivnica, CRO | 45.254809, 17.740955 | 120 | 4 Jul 2017 | CDC trap | Courtyard | 1 | 0 |
| 37 | Bzenica, CRO | 45.264149, 17.759133 | 127 | 5 Jul 2017 | Dipper | Ditch | 3 | 0 |
| 38 | Pleternica, CRO | 45.280330, 17.805730 | 113 | 5 Jul 2017 | BG-Sentinel | Tyre service | 2 | 0 |
| 39 | Velika, CRO | 45.473837, 17.649788 | 324 | 6 Jul 2017 | Dipper | Ditch | 1 | 0 |
| 40 | Samarske Stijene, CRO | 45.234964 14.953277 | 1198 | 10 Jul 2017 | CDC trap | Forest | 1 | 0 |
| 41 | Matić poljana, CRO | 45.288067, 14.892667 | 1032 | 10 Jul 2017 | CDC trap | Meadow | 1 | 0 |
| 42 | Tuk, CRO | 45.295389 14.887727 | 1007 | 10 Jul 2017 | CDC trap | Courtyard | 1 | 0 |
| 43 | Mrkopalj, CRO | 45.315706 14.849340 | 823 | 10 Jul 2017 | CDC trap | Courtyard | 1 | 0 |
| 44 | Sunger, CRO | 45.324525, 14.815220 | 799 | 10 Jul 2017 | CDC trap | Forest | 1 | 0 |
| 45 | Sungerski lug, CRO | 45.333999, 14.798857 | 802 | 10 Jul 2017 | CDC trap | Forest | 1 | 0 |
| 46 | Lokve, CRO | 45.356884, 14.747381 | 721 | 10 Jul 2017 | CDC trap | Courtyard | 1 | 0 |
| 47 | Mala Lešnica, CRO | 45.438975, 14.851273 | 326 | 10 Jul 2017 | CDC trap | Fishpond | 1 | 0 |
| 48 | Brod na Kupi, CRO | 45.462943, 14.859432 | 293 | 10 Jul 2017 | CDC trap | Courtyard | 1 | 0 |
| 49 | Belo Selo, CRO | 45.327922, 14.740807 | 819 | 12 Jul 2017 | CDC trap  Dipper | Backyard  Barrel | 1 trap  1 barrel | 0 |
| 50 | Gospić, CRO | 44.552612, 15.378811 | 563 | 31 Jul 2017 | BG-Sentinel | Tyre service | 2 | 0 |
| 51 | Bogdanica, CRO | 44.542755, 15.372151 | 564 | 31 Jul 2017 | CDC trap | Courtyard | 1 | 0 |
| 52 | Brušane 1, CRO | 44.502507, 15.264514 | 592 | 31 Jul 2017 | CDC trap | Courtyard | 1 | 0 |
| 53 | Trnovačko Novo Selo, CRO | 44.511057, 15.304314 | 562 | 31 Jul 2017 | CDC trap | Courtyard | 1 | 0 |
| 54 | Lički Novi, CRO | 44.512323, 15.317947 | 559 | 31 Jul 2017 | CDC trap | Farm | 1 | 0 |
| 55 | Dabarska Kosa, CRO | 44.556483, 15.116751 | 922 | 31 Jul 2017 | CDC trap | Forest | 1 | 0 |
| 56 | Ravni Dabar 1, CRO | 44.563246, 15.124822 | 846 | 31 Jul 2017 | CDC trap | Meadow | 1 | 0 |
| 57 | Ledenik, CRO | 44.534023, 15.112987 | 675 | 31 Jul 2017 | BG-Sentinel | Cemetery | 2 | 0 |
| 58 | Ledenik Cesarički, CRO | 44.542884, 15.103637 | 604 | 31 Jul 2017 | CDC trap | Courtyard | 1 | 0 |
| 59 | Baške Oštarije, CRO | 44.524235, 15.180549 | 933 | 1 Aug 2017 | CDC trap  Dipper | Courtyard  Barrel | 1 trap  3 barrels | 0 |
| 60 | Cesta Karlobag, CRO | 44.527028, 15.115167 | 605 | 1 Aug 2017 | Dipper | Sawmill | 5 | 0 |
| 61 | Brušane 2, CRO | 44.501229, 15.269197 | 585 | 1 Aug 2017 | Dipper | Barrel | 2 | 0 |
| 62 | Ravni dabar 2, CRO | 44.562859, 15.123073 | 785 | 2 Aug 2017 | CDC trap  Dipper | Courtyard  Barrel | 1 trap  3 barrels | 0 |
| 63 | Štirovača, CRO | 44.653468, 15.066612 | 1185 | 2 Aug 2017 | CDC trap  Dipper | Flooded forest  Pools and ponds | 1 trap  2 pools 1 pond | 0 |
| 64 | Novi Sad, SRB | 45.258871, 19.818778 | 80 | 06 Aug – 07 Dec 2018 *** | Ovitrap | Urban area  Car service | 4  1 | 0 |
| 65 | Šid 1, SRB | 45.155599, 19.175042 | 86 | 04 Aug – 28 Oct 2018 *** | Ovitrap | Border crossing | 5 | 0 |
| 66 | Sot, SRB | 45.172676, 19.348060 | 226 | 04 Aug – 28 Oct 2018 *** | Ovitrap | Border crossing | 5 | 0 |
| 67 | Neštin, SRB | 45.216704, 19.433094 | 125 | 04 Aug – 28 Oct 2018 *** | Ovitrap | Border crossing | 5 | 0 |
| 68 | Bačka Palanka, SRB | 45.239656, 19.398690 | 78 | 04 Aug – 28 Oct 2018 *** | Ovitrap | Border crossing | 5 | 0 |
| 69 | Bogojevo, SRB | 45.529249, 19.087853 | 78 | 04 Aug – 28 Oct 2018 *** | Ovitrap | Border crossing | 5 | 0 |
| 70 | Bezdan, SRB | 45.844697, 18.866679 | 83 | 04 Aug – 28 Oct 2018 *** | Ovitrap | Border crossing | 5 | 0 |
| 71 | Batrovci, SRB | 45.047225, 19.106996 | 80 | 19 May – 16 Oct 2018 *** | Ovitrap | Border crossing | 10 | 0 |
| 72 | Šid 2, SRB | 45.048508,19.192235 | 79 | 19 May – 16 Oct 2018 *** | Ovitrap | Toll station on highway | 5 | 0 |
| 73 | Ruma, SRB | 44.965209, 19.809962 | 91 | 19 May – 16 Oct 2018 *** | Ovitrap | Toll station on highway | 5 | 0 |
| 74 | Šid 3, SRB | 45.048426, 19.212162 | 80 | 19 May – 16 Oct 2018 *** | Ovitrap | Petrol station on highway | 5 | 0 |
| 75 | Adaševci, SRB | 45.035033, 19.449694 | 82 | 19 May – 16 Oct 2018 *** | Ovitrap | Petrol station in highway | 5 | 0 |
| 76 | Lesenica, SRB | 44.644389, 19.288959 | 103 | 06 Aug – 29 Sept 2018 *** | Ovitrap | Petrol station | 10 | 0 |
| 77 | Loznica, SRB | 44.531991, 19.202970 | 119 | 21 Aug – 15 Sept 2018 *** | Ovitrap | City center | 10 | 0 |
| 78 | Banja Koviljača, SRB | 44.495798, 19,135612 | 127 | 21 Aug – 15 Sept 2018 *** | Ovitrap | Urban area | 10 | 0 |
| 79 | Bosanski Šamac, BiH | 45.060728, 18.470338 | 87 | 07 Jul – 03 Oct 2017 *** | Ovitrap | Cemetery  Tyre service  Courtyard | 5  1  2 | 0 |
| 80 | Orašje, BiH | 45.037610, 18.693681 | 84 | 07 Jul – 03 Oct 2017 *** | Ovitrap | Courtyard  Border crossing  Cemetery  Car service | 2  1  1  1 | 0 |
| 81 | Modriča, BiH | 44.957444, 18.302815 | 105 | 07 Jul – 03 Oct 2017 *** | Ovitrap | Courtyard  Cemetery  Petrol station | 3  1  1 | 0 |

* Ovitraps operated continuously 04 Aug – 28 Oct 2018, one positive 21 Aug 2018, other positive 15 Sept 2018.

** Ovitraps operated continuously 07 Jul – 03 Oct 2017

*** Ovitraps operated continuously

^#^ for specimens subjected to population genetic analyses see Table 2 (several larvae were processed for permanent microscopic slides and were not available for genetic analyses)
